# Supplementary material for: Exploring Factors Influencing Patient Delay Behavior in Oral Cancer: The Development of a Risk Prediction Model in Western China
Source: Healthcare (Basel). 2024 Nov 12;12(22):2252. doi: 10.3390/healthcare12222252 (PMC11593436; doi:10.3390/healthcare12222252)
Supplement: Supplementary file 1 [file healthcare-12-02252-s001.zip › healthcare-3185604-supplementary.pdf]

Exploring Factors Influencing Patient Delay Behavior in Oral Cancer: The Development of a Risk Prediction Model in Western China

Yuanyuan Yang [1,2,†], Huan Ning [2,†], Bohui Liang [3,†], Huaming Mai [1], Jie Zhou [2], Jing Yang [2] and Jiegang Huang [2,4,*]

Table S1. Single factor analysis of delayed medical attention for oral cancer patients in western China

Variable	Value	During the strict travel restrictions						lift restrictions					
		Patient without delay in seeking healthcare(%)		Patient with delay in seeking healthcare(%)		Statistics	P	Patient without delay in seeking healthcare(%)		Patient with delay in seeking healthcare(%)		Statistics	P
Gender	Male	57	66.3	73	59.8	0.893	0.345	93	69.4	95	62.1	1.69	0.194
	Female	29	33.7	49	40.2			41	30.6	58	37.9		
Nation	Han Nationality	49	57.0	70	57.4	0.003	0.954	81	60.4	92	60.1	0.003	0.956
	Ethnic minorities	37	43.0	52	42.6			53	39.6	61	39.9		
Occupation	Workers	18	20.9	23	18.9	2.451	0.302	39	29.1	46	30.1	0.791	0.673
	Farmers	31	36.0	57	46.7			43	32.1	55	35.9		
	Professionals and others	37	43.0	42	34.4			52	38.8	52	34.0		
Age	＜40	12	14.0	5	4.1	4.025	0.045	21	15.7	20	13.1	0.025	0.874
	40-59	41	47.7	58	47.5			57	42.5	70	45.8		
	＞60	33	38.4	59	48.4			56	41.8	63	41.2		
Residency	Rural	37	43.0	67	54.9	2.298	0.130	67	50.0	82	53.6	0.370	0.543
	Urban	49	57.0	55	45.1			67	50.0	71	46.4		

Educational level	Below primary education	5	5.8	6	4.9	1.300	0.254	13	9.7	16	10.5	0.533	0.465
	primary school	12	14.0	31	25.4			29	21.6	35	22.9		
	Middle school	31	36.0	34	27.9			37	27.6	49	32.0		
	high school	19	22.1	31	25.4			33	24.6	29	19.0		
	≥College	19	22.1	20	16.4			22	16.4	24	15.7		
Monthly income	<1500 RMB	17	19.8	32	26.2	3.787	0.052	40	29.9	59	38.6	1.924	0.165
	1500-3000 RMB	19	22.1	38	31.1			33	24.6	30	19.6		
	3001-5000 RMB	30	34.9	32	26.2			36	26.9	45	29.4		
	5001-8000 RMB	16	18.6	15	12.3			21	15.7	13	8.5		
	>8000 RMB	4	4.7	5	4.1			4	3.0	6	3.9		
Marital status	Single	6	7.0	3	2.5	3.712	0.156	16	11.9	14	9.2	0.594	0.743
	Married	76	88.4	108	88.5			101	75.4	119	77.8		
	Others	4	4.7	11	9.0			17	12.7	20	13.1		
Live with others	Yes	80	93.0	117	95.9	0.834	0.368	120	89.6	138	90.2	0.033	0.857
	No	6	7.0	5	4.1			14	10.4	15	9.8		
Person who lives with	Spouse	27	31.4	94	77.0	45.393	**0.000**	28	20.9	109	71.2	83.862	**0.000**
	Adult Children	52	60.5	21	17.2			81	60.4	20	13.1		
	Others	7	8.1	7	5.7			25	18.7	24	15.7		
Medical staff friends/relatives	Yes	44	51.2	69	56.6	0.592	0.442	65	48.5	55	35.9	4.632	**0.031**
	No	42	48.8	53	43.4			69	51.5	98	64.1		
Medical insurance type	Basic medical insurance system for town staff	36	41.9	33	27.0	5.051	0.08	44	32.8	49	32.0	0.165	0.921
	New Cooperative Medical System (NCMS)	44	51.2	77	63.1			78	58.2	92	60.1		

	Urban Residents Basic Health Insurance	6	7.0	12	9.8			12	9.0	12	7.8		
Systemic disease type	Circulation system disease	27	31.4	37	30.3	2.146	0.543	31	23.1	38	24.8	0.680	0.878
	Endocrine system disease	7	8.1	5	4.1			10	7.5	13	8.5		
	Others	17	19.8	31	25.4			37	27.6	36	23.5		
	No systemic disease	35	40.7	49	40.2			56	41.8	66	43.1		
Follow-up status of systemic disease	Never follow up	6	7.0	16	13.1	5.851	0.119	21	15.7	30	19.6	4.181	0.243
	Irregular follow up	25	29.1	42	34.4			30	22.4	40	26.1		
	regular follow up	20	23.3	15	12.3			28	20.9	19	12.4		
	No systemic disease	35	40.7	49	40.2			55	41.0	64	41.8		
Sites inside the mouth	Buccal division	10	11.6	31	25.4	10.338	**0.035**	21	15.7	22	14.4	6.732	0.151
	Tongue	50	58.1	48	39.3			71	53.0	70	45.8		
	Gums	9	10.5	21	17.2			17	12.7	37	24.2		
	Palatine	5	5.8	6	4.9			7	5.2	9	5.9		
	Face and lips/parotid gland/floor of mouth/throat	12	14.0	16	13.1			18	13.4	15	9.8		
First visit institution	Clinics	18	20.9	33	27.0	4.558	0.336	28	20.9	40	26.1	3.052	0.549
	Primary health care facilities	8	9.3	17	13.9			15	11.2	20	13.1		
	Secondary care facilities	18	20.9	30	24.6			23	17.2	31	20.3		
	Tertiary health care facilities	33	38.4	33	27.0			48	35.8	44	28.8		
	Dental hospitals	9	10.5	9	7.4			20	14.9	18	11.8		
Type of	Antibiotic	18	20.9	36	29.5	5.103	0.277	36	26.9	52	34.0	4.569	0.334

self-medication	Traditional Chinese medicine	5	5.8	12	9.8			11	8.2	10	6.5		
	Anti-ulcer drugs/Topical drugs	11	12.8	16	13.1			14	10.4	22	14.4		
	Painkillers and others	2	2.3	5	4.1			5	3.7	8	5.2		
	No self-medication	50	58.1	53	43.4			68	50.7	61	39.9		
Impact of COVID-19	Yes	18	20.9	74	60.7	10.052	**0.002**	22	16.4	50	32.7	7.896	**0.005**
	No	68	79.1	48	39.3			112	83.6	103	67.3		
Smoking dosage	＜5 sticks/d	2	2.3	2	1.6	0.048	0.826	5	3.7	4	2.6	0.023	0.879
	5-10sticks/d	3	3.5	7	5.7			4	3.0	8	5.2		
	11-15sticks/d	6	7.0	4	3.3			8	6.0	4	2.6		
	16-20sticks/d	3	3.5	6	4.9			4	3.0	10	6.5		
	＞20sticks/d	21	24.4	32	26.2			35	26.1	37	24.2		
	No smoking	51	59.3	71	58.2			78	58.2	90	58.8		
Second-hand smoking	Yes	46	53.5	51	41.8	0.454	0.500	57	42.5	85	55.6	4.843	0.028
	No	40	46.5	71	58.2			77	57.5	68	44.4		
Alcohol consumed	＜50ml/d	2	2.3	3	2.5	1.677	0.195	13	9.7	12	7.8	0.048	0.826
	50-100ml/d	2	2.3	5	4.1			6	4.5	15	9.8		
	101-150ml/d	1	1.2	6	4.9			2	1.5	7	4.6		
	151-200ml/d	4	4.7	2	1.6			2	1.5	6	3.9		
	＞200ml/d	19	22.1	35	28.7			29	21.6	24	15.7		
	No drinking	58	67.4	71	58.2			82	61.2	89	58.2		
Areca-nut	Yes	9	10.5	12	9.8	0.022	0.882	11	8.2	22	14.4	2.673	0.102
	No	77	89.5	110	90.2			123	91.8	131	85.6		
HL1	Very difficult/Difficult	8	9.3	19	15.6	10.409	**0.001**	7	5.2	22	14.4	9.537	**0.002**

	Relatively difficult/Somewhat difficult	5	5.8	26	21.3			17	12.7	29	19.0		
	No difficulty at all	73	84.9	77	63.1			110	82.1	102	66.7		
HL2	Very difficult	1	1.2	5	4.1	20.118	0.000	9	6.7	5	3.3	10.989	0.001
	Difficult	5	5.8	13	10.7			13	9.7	15	9.8		
	Relatively difficult	4	4.7	16	13.1			9	6.7	26	17.0		
	Somewhat difficult	14	16.3	40	32.8			23	17.2	57	37.3		
	No difficulty at all	62	72.1	48	39.3			80	59.7	50	32.7		
HL3	Very difficult	3	3.5	22	18.0	20.427	0.000	12	9.0	23	15.0	19.991	0.000
	Difficult	5	5.8	11	9.0			8	6.0	11	7.2		
	Relatively difficult	11	12.8	21	17.2			18	13.4	38	24.8		
	Somewhat difficult	31	36.0	48	39.3			39	29.1	57	37.3		
	No difficulty at all	36	41.9	20	16.4			57	42.5	24	15.7		
HL	Score	13.19±2.61		11.30±3.21		-5.339	0.000	12.67±3.06		11.40±3.09		-5.070	0.000
Heath literacy level	Inadequate	10	11.6	33	27.0	7.315	0.007	24	17.9	33	21.6	0.601	0.438
	Adequate	76	88.4	89	73.0			110	82.1	120	78.4		
Access to knowledge	Relatives and friends	52	60.5	60	49.2	10.741	0.005	57	42.5	72	47.1	0.623	0.732
	Hospital medical staff	32	37.2	42	34.4			58	43.3	60	39.2		
	Network and others	2	2.3	20	16.4			19	14.2	21	13.7		
Presumed cause	Smoking/Second-hand smoke/Alcohol consumption	16	18.6	20	16.4	4.430	0.351	24	17.9	17	11.1	6.316	0.177
	Areca-nut chewing	3	3.5	4	3.3			3	2.2	8	5.2		

	Missing teeth/Stimulation of dentures	21	24.4	39	32.0			36	26.9	37	24.2		
	Genetic factors and others	19	22.1	15	12.3			27	20.1	26	17.0		
	No idea	27	31.4	44	36.1			44	32.8	65	42.5		
Checking mouth regularly	Yes	13	15.1	3	2.5	11.381	0.001	17	12.7	2	1.3	14.963	0.000
	No	73	84.9	119	97.5			117	87.3	151	98.7		
Chew hard objects	Yes	32	37.2	60	49.2	2.930	0.087	53	39.6	88	57.5	9.224	0.002
	No	54	62.8	62	50.8			81	60.4	65	42.5		
Brushing frequency	≥2/d	58	67.4	51	41.8	14.406	0.000	90	67.2	64	41.8	19.561	0.000
	1/d	24	27.9	53	43.4			37	27.6	67	43.8		
	No brushing or not sure	4	4.7	18	14.8			7	5.2	22	14.4		
Distance of nearest medical facility	＜2km	46	53.5	42	34.4	12.060	0.001	73	54.5	57	37.3	10.079	0.001
	2-5km	34	39.5	48	39.3			42	31.3	55	35.9		
	6-10km	3	3.5	23	18.9			11	8.2	29	19.0		
	10-15km	2	2.3	7	5.7			7	5.2	6	3.9		
	＞15km	1	1.2	2	1.6			1	0.7	6	3.9		
Vehicle	Walking	16	18.6	33	27.0	18.714	0.000	28	20.9	38	24.8	17.155	0.001
	Electric vehicles	45	52.3	51	41.8			57	42.5	61	39.9		
	Cars	22	25.6	14	11.5			40	29.9	23	15.0		
	Public transport and others	3	3.5	24	19.7			9	6.7	31	20.3		
Accessibility of dental hospitals	Poor/Relatively poor	67	77.9	101	82.8	0.773	0.379	104	77.6	117	76.5	0.053	0.819
	Good	5	5.8	6	4.9			11	8.2	10	6.5		
	Very good	14	16.3	15	12.3			19	14.2	26	17.0		

Accessibility of primary health care facilities	Poor/Relatively poor	3	3.5	5	4.1	0.000	0.983	3	2.2	7	4.6	0.326	0.568
	Good	12	14.0	16	13.1			16	11.9	18	11.8		
	Very good	71	82.6	101	82.8			115	85.8	128	83.7		
Accessibility of secondary care facilities	Poor	4	4.7	17	13.9	0.309	0.578	10	7.5	19	12.4	2.275	0.131
	relatively poor	13	15.1	10	8.2			16	11.9	22	14.4		
	Good	14	16.3	19	15.6			29	21.6	33	21.6		
	Very good	55	64.0	76	62.3			79	59.0	79	51.6		
Accessibility of tertiary health care facilities	Poor	31	36.0	53	43.4	1.999	0.157	49	36.6	57	37.3	0.005	0.943
	relatively poor	14	16.3	19	15.6			14	10.4	16	10.5		
	Good	5	5.8	13	10.7			15	11.2	16	10.5		
	Very good	36	41.9	37	30.3			56	41.8	64	41.8		
Accessibility to dental clinics	Poor	23	26.7	44	36.1	2.533	0.112	40	29.9	48	31.4	0.369	0.544
	relatively poor	9	10.5	13	10.7			11	8.2	16	10.5		
	Good	9	10.5	14	11.5			19	14.2	22	14.4		
	Very good	45	52.3	51	41.8			64	47.8	67	43.8		
